# *Myc* Dynamically and Preferentially Relocates to a Transcription Factory Occupied by *Igh*

**DOI:** 10.1371/journal.pbio.0050192

**Published:** 2007-07-10

**Authors:** Cameron S Osborne, Lyubomira Chakalova, Jennifer A Mitchell, Alice Horton, Andrew L Wood, Daniel J Bolland, Anne E Corcoran, Peter Fraser

**Affiliations:** Laboratory of Chromatin and Gene Expression, The Babraham Institute, Cambridge, United Kingdom

## Abstract

Transcription in mammalian nuclei is highly compartmentalized in RNA polymerase II-enriched nuclear foci known as transcription factories. Genes in *cis* and *trans* can share the same factory, suggesting that genes migrate to preassembled transcription sites. We used fluorescent in situ hybridization to investigate the dynamics of gene association with transcription factories during immediate early (IE) gene induction in mouse B lymphocytes. Here, we show that induction involves rapid gene relocation to transcription factories. Importantly, we find that the *Myc* proto-oncogene on Chromosome 15 is preferentially recruited to the same transcription factory as the highly transcribed *Igh* gene located on Chromosome 12. *Myc* and *Igh* are the most frequent translocation partners in plasmacytoma and Burkitt lymphoma. Our results show that transcriptional activation of IE genes involves rapid relocation to preassembled transcription factories. Furthermore, the data imply a direct link between the nonrandom interchromosomal organization of transcribed genes at transcription factories and the incidence of specific chromosomal translocations.

## Author Summary

Many different types of cancer result from gene translocations. Specifically, two different chromosomes can be joined that fuse growth control genes with powerful regulatory elements, leading to unrestricted control of cell growth. Translocation partner genes must physically encounter each other in the nucleus to undergo a translocation; how they find each other in the crowded nucleus is unknown. We showed previously that gene transcription occurs at a few hundred discrete nuclear sites called transcription factories. In the current study we investigated the effects of activation of the *Myc* proto-oncogene and examined its location with respect to transcription factories and its common translocation partner, the *immunoglobulin heavy chain (Igh)* gene. We found that switching on the *Myc* gene leads to its rapid relocation to a transcription factory. Surprisingly, we found that the activated *Myc* frequently chooses the same transcription factory as the highly transcribing *Igh* gene. This close juxtaposition of translocation partner genes at a shared transcription factory may provide the opportunity for a chromosomal translocation, and thus may be the first step in the genesis of several types of cancers.

## Abstract 3

Transcription in mammalian nuclei occurs in nuclear foci known as transcription factories. Genes from different chromosomes can share the same factory, suggesting that factories are preassembled and genes migrate to them.

## Introduction

Interphase chromosomes are organized in tissue-specific arrangements in nuclei, suggesting that chromosomal position and juxtaposition play a role in gene expression [1–5]. Nonrandom chromosome positioning has also been implicated in the frequency of specific chromosomal translocations. For example, Chromosomes 12 and 15, which contain the frequent B cell translocation partners *immunoglobulin heavy chain (Igh)* and the proto-oncogene *Myc,* are preferred neighbors in mouse splenic lymphocytes [3]. Similarly, in human lymphoid cells *MYC* and *IGH* are found in the same vicinity in about one-third of nuclei [6]. Any process that brings these genes together would obviously be expected to increase the risk of a translocation between them; however, almost nothing is known about the forces that organize chromosomes in the nucleus.

Nascent transcription occurs at RNA polymerase II (RNAPII)-rich nuclear foci known as transcription factories [7–11]. These sites are highly enriched in the hyperphosphoryated forms of RNAPII involved in transcription initiation and elongation [7,11]. Previous findings suggest that active mammalian genes are transcribed in bursts of activity punctuated by long periods of relative inactivity [9,12–14]. This concept is supported by recent live-cell studies showing that gene expression [15,16], and in particular gene transcription [17], occur in discrete pulses. We and others [9,10] have observed a virtually absolute correspondence between transcriptional activity at individual gene alleles and their positioning within transcription factories, whereas identical inactive alleles, often in the same cell, are clearly positioned away from factories. Collectively, these data could be interpreted to imply that the engagement of genes at factories is dynamic; however, they could equally be construed to indicate that a transcription factory nucleates around an individual gene during a transcriptional burst. Arguing against the latter interpretation is the finding that multiple genes in *cis* and *trans* can frequently share the same factory, which strongly suggests that genes migrate to preassembled transcription sites for transcription [9].

In this study, we investigated the positioning of immediate early (IE) genes relative to transcription factories and other B cell expressed genes during IE induction. We found that before activation the majority of IE alleles are not associated with transcription factories, whereas upon induction, IE genes rapidly relocate to preformed transcription factories. Remarkably, we observed preferential recruitment of the proto-oncogene *Myc* to the same transcription factory that is occupied by its frequent translocation partner, *Igh*. Our results suggest that this frequent and preferential juxtaposition may provide the opportunity for a chromosomal translocation, and may in part dictate the incidence with which specific chromosomal translocations occur.

## Results

### Transcriptional Frequencies of IE Gene Alleles Vary during Induction

**Figure 1:**
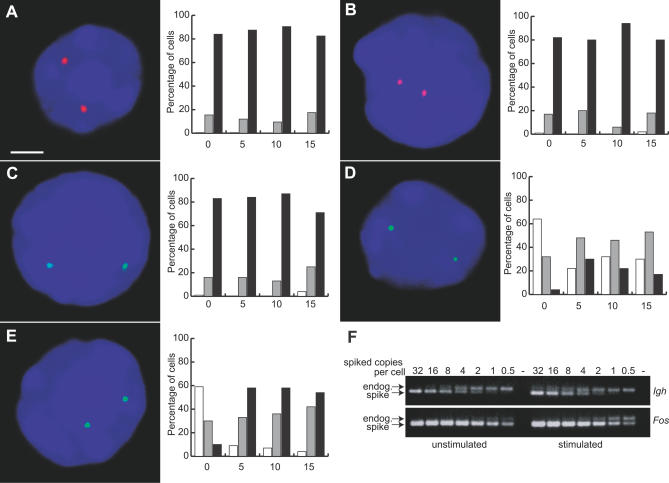
RNA FISH and Transcription Analysis in Unstimulated and Stimulated B Cells RNA FISH in splenic B cells for *Igh* (red) (A), *Igk* (red) (B), *Igl* (green) (C), *Fos* (green) (D), and *Myc* (green) (E) gene transcription. DAPI staining is blue. Scale bar, 2 μm. We have shown nuclei in which both signals are in the same optical plane. The graphs show the percentage of cells with zero (white), one (grey), or two (black) transcription signals by RNA FISH in B cells, unstimulated and stimulated for the times indicated (in minutes). Results shown are from a single experiment, which was repeated three times, with similar results in every case. One hundred nuclei were assessed for each time point. The percentage of transcribing alleles was significantly different between unstimulated and stimulated cells for both *Fos* (0 min versus 5 min, *p* = 4.0 × 10^−12^; versus 10 min, *p* = 1.3 × 10^−7^; and versus 15 min, *p* = 6.3 × 10^−7^) and *Myc* (0 min versus 5 min, 3.9 × 10^−23^; versus 10 min, 1.1 × 10^−22^; and versus 15 min, 1.1 × 10^−22^). (F) RT-PCR analysis with serial dilutions of spiked competitor to determine absolute nascent transcript numbers for *Igh* and *Fos* in unstimulated and stimulated B cells. The amount of competitor RNA spiked into each RNA extraction is shown above. The – lane is the no-DNA PCR control.

Resting B cells can be stimulated through the B cell receptor signaling pathway to rapidly increase transcription and mRNA expression of the IE genes *Fos* and *Myc* [18]. We used RNA fluorescent in situ hybridization (FISH) with gene-specific intron probes to investigate the transcriptional activity of several genes during IE gene induction in mouse B lymphocytes (Figure 1). We found that transcription frequencies vary for several genes in B cells, similar to our previous observations in erythroid cells [9]. Transcription signals for the B cell-specific gene *Igh* are present at approximately 90% of alleles both before and after induction (Figure 1A). In the vast majority of cells both alleles are actively transcribed (>80% of cells), whereas a smaller percentage of cells have only single signals, consistent with our previous findings [19]. The immunoglobulin light chain genes *Igk* and *Igl* also have transcription signals at approximately 90% of alleles, with approximately 80% of cells having two transcription signals (Figure 1B and 1C). These data show constitutive transcriptional activity at nearly all alleles that is unchanged upon IE induction. In contrast, transcription of the IE proto-oncogenes *Fos* and *Myc* is significantly lower in unstimulated cells, with 20% and 26% of alleles, respectively, displaying transcription signals (Figure 1D and 1E). Most cells (~60%) have two silent alleles, some cells have one active allele (~30%), and a minority of cells have two active alleles (<10%). Upon induction, the percentage of loci with transcription signals for *Fos* and *Myc* rises dramatically within 5 min to 53% and 75%, respectively. This rise is the result of a dramatic increase in the percentage of cells with two active alleles and, to a lesser extent, an increase in cells with one active allele. The fold increases in the percentage of active alleles are in precise agreement with run-on transcription studies in mouse B cells that demonstrated a 2- to 3-fold induction of *Fos* and *Myc* transcription in stimulated cells [18]. These results suggested the possibility that increased IE expression could be accounted for by transcriptional recruitment of additional IE alleles rather than an increase in the “basal” rate of transcription at all IE alleles.

### IE Gene Induction Results from Activation of Previously Inactive Alleles

We questioned whether the increase in nascent transcription levels seen in run-ons could be accounted for solely by the transcriptional recruitment of additional IE alleles. We used a sensitive reverse transcription PCR (RT-PCR) technique capable of quantitating the average absolute number of primary transcripts per cell [20]. In this method serial dilutions of a known amount of a spiked competitor RNA that contains a small internal deletion is compared to the amount of endogenous primary transcript in total RNA preps from a known number of cells. We adapted this method by using PCR primers that flank a 5′ splice donor site. Cleavage of the primary transcript at the 5′ donor site occurs soon after the RNA polymerase has passed the 3′ splice acceptor site, at the end of the intron sequence [21]. Quantitative detection of RT-PCR products from this part of the primary transcript provides an extremely sensitive measure of the number of transcripts being synthesized over that intron. An estimate of the average number of primary transcripts per gene can be calculated by extrapolating to the full size of the transcription unit.

We found that the average number of *Igh* primary transcripts does not change during B cell induction (Figure 1F; Table 1), consistent with our FISH data, which indicate that *Igh* transcription is unaffected by stimulation. We detected approximately 4.3 and 4.5 unspliced primary transcripts on the *Igh* intron per cell in unstimulated and stimulated cells, respectively. As this intron is approximately half the *Igh* transcription unit length, we calculated that there are an average of 8.5 and 9.0 primary transcripts being produced from the two *Igh* transcription units per cell (4.3 and 4.5 per *Igh* allele). Since RNA FISH shows that 90% of *Igh* alleles are transcriptionally active, we estimate that there are approximately five transcripts being produced on each active allele.

**Table 1:**
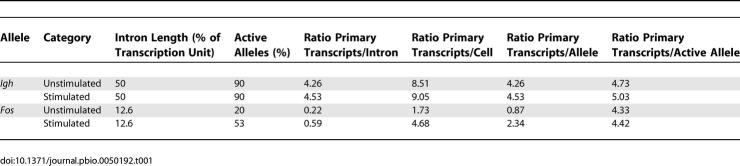
Quantitation of Primary Transcripts on *Igh* and *Fos* Alleles

The picture for the IE *Fos* gene is very different. In unstimulated cells we found that the extrapolated, average absolute number of *Fos* primary transcripts per cell is 1.73 copies. This is less than one primary *Fos* transcript per allele, indicating that not all *Fos* alleles are transcriptionally active in unstimulated cells. If we calculate the average number of *Fos* transcripts per active allele based on our RNA FISH data in which 20% of *Fos* alleles showed a transcription signal, we arrive at an average number of 4.3 *Fos* primary transcripts per active allele. In stimulated cells *Fos* primary transcript intron copies per cell increase approximately 2.7-fold, consistent with the 2.65-fold increase in the percentage of actively transcribed *Fos* alleles determined by RNA FISH. Comparison of the number of *Fos* primary transcripts per active allele indicates that the number of *Fos* transcripts per active allele does not change upon induction (4.3 versus 4.4 copies). These data show that our RNA FISH technique is very sensitive and is capable of measuring very small numbers of primary transcripts at a transcription site. In addition, these results show that when we do not see an RNA FISH signal over a particular allele, it is truly “off” and has no primary transcripts associated with it. Collectively, these results show that *Fos* IE gene induction occurs via transcriptional activation of additional, previously inactive alleles, rather than by simply increasing the “basal” rate of transcription of all alleles.

### IE Genes Rapidly Associate with Transcription Factories upon Induction

Our previous studies in erythroid cells suggested that gene association with transcription factories is dynamic, with genes moving to preformed factories in order to transcribe [9]. Inducible gene expression in B cells permitted us to examine the dynamics of transcription in relation to transcription factories. We speculated that the activation of previously quiescent IE alleles upon B cell induction may involve repositioning of alleles to transcription factories. However, the *Myc* gene has a well-characterized attenuation site that is thought to block passage of RNAP II, resulting in a stalled polymerase [22]. This observation leaves open the possibility that silent *Myc* alleles may be pre-positioned in factories awaiting removal of a transcriptional block.

**Figure 2:**
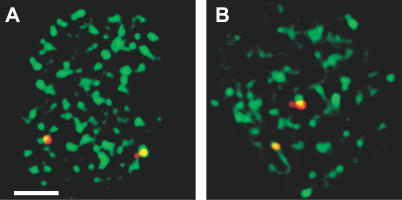
Transcription of *Myc* and *Igh* Occurs at Transcription Factories in B Cells RNA immuno-FISH (RNA FISH combined with immunodetection of RNAP II) shows the positions of transcribing *Myc* (A) and *Igh* (B) alleles (red) relative to transcription factories (green). Shown are deconvoluted, single optical sections of stimulated B cells. We have presented cells in which both transcribing alleles are in the same focal plane. The *Myc* and *Igh* RNA FISH signals show 92% (*n* = 52) and 90% (*n* = 89) co-association with RNAP II foci, respectively. Scale bar, 2 μm.

We first examined the positions of actively transcribed genes relative to transcription factories, using RNA immuno-FISH (Figure 2). We found that 92% of *Myc* RNA FISH signals are associated with strong RNAP II foci (Figure 2A). Similarly, 90% of transcriptionally active *Igh* alleles are associated with strongly staining RNAP II foci (Figure 2B), consistent with previous observations of erythroid-expressed genes [9,10]. Others have shown that the remaining 10% of RNA FISH signals localize to weakly staining RNAPII foci [10], indicating that essentially all transcriptionally active alleles associate with transcription factories. This observation agrees with previous studies that showed a good correspondence between pulse-labeled nascent RNA and RNAPII foci [7,11]. We conclude that all *Myc* and *Igh* transcription occurs at transcription factories.

**Figure 3:**
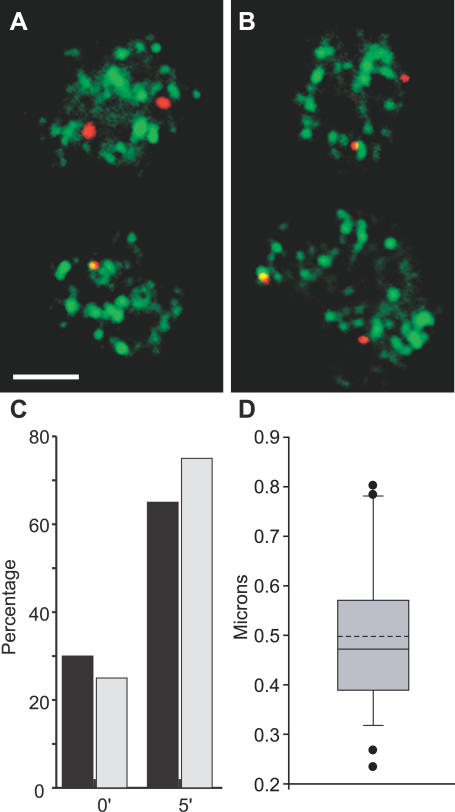
Relocation of *Myc* Alleles to RNAP II Foci upon B Cell Activation (A and B) DNA immuno-FISH of *Myc* locus (red) with RNAP II staining (green) in unstimulated (A) and stimulated (B) splenic B cells. Deconvoluted, single optical sections of the nuclei are shown. Scale bar, 2 μm. (C) Comparison of the percentage of *Myc* loci that overlap with an RNAP II focus by DNA immuno-FISH (black, *n* = 81 and *n* = 88 for unstimulated and stimulated, respectively), and the percentage of transcriptionally active *Myc* alleles by RNA FISH (gray) in unstimulated (0′) and 5 min–stimulated (5′) B cells. Comparison of DNA immuno-FISH results in unstimulated versus stimulated cells, *p* = 6.6 × 10^−6^. Comparison of RNA FISH results in unstimulated versus stimulated cells, *p* = 3.9 × 10^−23^. Comparisons between DNA immuno-FISH and RNA FISH results in unstimulated and stimulated cells yielded *p*-values of 0.55 and 0.12, respectively. (D) Box and whiskers plot of the distribution of 3D measurements of the separation distance between non-RNAP II-associated *Myc* alleles and the nearest RNAP II focus by DNA immuno-FISH. Lower and upper whiskers denote the 10th and 90th percentiles, respectively, of the distribution. The lower and upper limits of the boxes indicate the 25th and 75th percentiles, respectively. Solid and dashed lines in the box denote the median and mean, respectively. Outliers are shown as filled circles.

Next, we investigated the position of nontranscribing alleles by DNA immuno-FISH, which detects DNA of both active and inactive alleles and RNAP II proteins. We found that approximately 30% of *Myc* DNA FISH signals overlapped with RNAP II foci in unstimulated cells, while 70% were not associated with RNAP II foci. These results are consistent with the percentage of transcriptionally active alleles detected by RNA FISH (Figure 3A and 3C), and show that the inactive *Myc* alleles are not associated with transcription factories, but are instead positioned away from these sites. Three-dimensional DNA FISH measurements between the inactive *Myc* alleles and the nearest transcription factory show that on average silent *Myc* alleles are 500 nm from the nearest factory (Figure 3D).

After 5 min of stimulation the percentage of *Myc* loci associated with transcription factories increased to 65%, in agreement with the increased percentage of actively transcribing *Myc* alleles determined by RNA FISH (Figure 3B and 3C). These results show that *Myc* induction involves an increase in the percentage of *Myc* alleles associated with transcription factories. The increase of gene association with factories could be achieved in two ways. *Myc* transcriptional induction could involve the nucleation of transcription factories on newly activated *Myc* alleles. Alternatively, transcriptional induction could involve the rapid relocation of silent *Myc* alleles to preassembled transcription factories.

### IE Genes Relocate to Preexisting Transcription Factories

**Figure 4:**
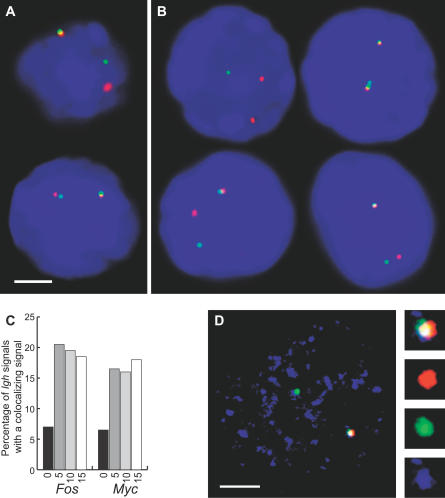
Colocalization of IE Gene Transcription Signals with *Igh* (A and B) Double-label RNA FISH in stimulated splenic B cells for *Igh* (red) and *Fos* (green) (A) or *Igh* (red) and *Myc* (green) (B). Scale bar, 2 μm. (C) The percentages of *Igh* signals with visibly overlapping (colocalizing) *Fos* or *Myc* signals, before and after B cell stimulation for the times indicated in minutes. One hundred nuclei were assessed for signals in each case. There are significantly more colocalizing signals in stimulated cells compared to unstimulated for *Fos* (0 min versus 5 min, *p* = 1.2 × 10^−4^; versus 10 min, *p* = 3.3 × 10^−4^; and versus 15 min, 1.4 × 10^−3^) and *Myc* (0 min versus 5 min, *p* = 2.5 × 10^−3^; versus 10 min, 4.2 × 10^−3^; and versus 15 min, *p* = 1.9 × 10^−4^). (D) A single optical section of a triple-label, RNA immuno-FISH on stimulated B cells showing *Igh* (red) and *Myc* (green) transcription, and RNAPII foci (blue). Images on right are enlargements of colocalized transcription signals associating with the same RNAPII focus. Scale bar, 2 μm.

The synchronous induction of IE gene alleles described above allowed us to directly test these alternate scenarios. The *Igh* locus on mouse Chromosome 12 is positioned 28 Mb telomeric to the *Fos* locus. Approximately 90% of *Igh* alleles exhibit RNA FISH signals in B cells (Figure 1A) [19], and are associated with transcription factories, indicating that the *Igh* locus undergoes nearly constant transcription, similar to the highly expressed *Hbb* locus in erythroid cells [9,23]. We therefore used *Igh* RNA FISH signals as factory reference points and scored the percentage of *Igh* transcription signals that have a colocalizing (overlapping) *Fos* signal, before and after induction using double-label RNA FISH. In unstimulated cells approximately 7% of *Igh* signals had a colocalizing *Fos* signal (Figure 4A and 4C). After induction, colocalization nearly tripled, with 20% of *Igh* signals having a colocalizing *Fos* signal. These results suggest that a significant proportion of the newly activated *Fos* alleles move to a factory that is already occupied by an *Igh* allele rather than forming their own factory.

### Frequent Translocation Partner Genes Often Share the Same Transcription Factory

We previously showed a low but significant level of interchromosomal associations between the highly transcribed *Hbb* and *Hba* genes in erythroid cells [9]. We hypothesized that the preferred neighbor arrangement of Chromosomes 12 and 15 in B cells [3] might allow *Myc* and *Igh* to co-associate with the same transcription factory in *trans*. Using double-label RNA FISH as above, we found that approximately 6% of *Igh* signals have a colocalizing *Myc* signal in unstimulated cells (Table 2). Comparing the percentage of active *Myc* alleles that colocalized with an *Igh* signal to those that did not, we found that a remarkable 25% of the transcribing *Myc* alleles colocalized with *Igh* in *trans* prior to induction. Upon induction, as we observed a 2.9-fold increase in the percentage of transcribing *Myc* alleles, we found a 2.5-fold increase in the percentage of *Igh* alleles with a colocalizing *Myc* signal (Figure 4B and 4C; Table 2). Again, by comparing colocalizing versus noncolocalizing *Myc* signals we found that approximately one-fourth of the active *Myc* alleles (22–24%; Table 2) were associated with *Igh* alleles upon induction. Thus, one-fourth of the newly activated *Myc* alleles, which were previously located away from transcription factories, had moved to a factory occupied by *Igh*. We confirmed that colocalizing *Myc* and *Igh* transcription signals co-associated with a shared transcription factory using triple-label RNA immuno-FISH to detect transcriptionally active *Myc* and *Igh* alleles and RNAP II foci (Figure 4D). We found that all colocalizing *Myc* and *Igh* signals overlapped with the same transcription factory.

**Table 2:**
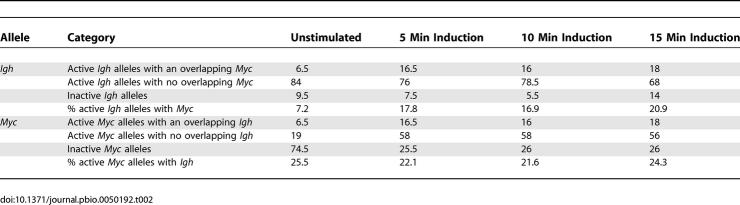
Percentage of Transcribing and Colocalizing *Igh* and *Myc* Alleles

**Figure 5:**
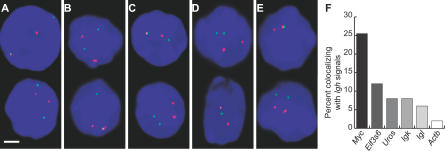
Trans Colocalization of Transcriptionally Active Genes Double-label RNA FISH in unstimulated B cells to detect *Igh* (red) and (A) *Eif3s6* (green), (B) *Uros* (green), (C) *Igk* (green), (D) *Igl* (green), and (E) and *Actb* (green). DAPI staining is blue. Scale bar, 2 μm. (F) The percentage of *Myc, Eif3s6, Uros, Igk, Igl,* and *Actb* signals that overlap with an *Igh* signal. One hundred nuclei were assessed for each case except *Uros,* for which 70 nuclei were examined. *Myc*-*Igh* versus *Eif3s6*-*Igh, p* = 0.02; versus *Uros*-*Igh, p* = 0.01; versus *Igk-Igh, p* = 5.9 × 10^−4^; versus *Igl-Igh, p* = 5.2 × 10^−5^; and versus *Actb*-*Igh, p* = 1.0 × 10^−6^.

In order to put this extraordinarily high frequency of interchromosomal *Myc*-*Igh* colocalization into perspective we compared the colocalization frequencies between *Igh* and five other B cell-expressed genes. One gene, *Eif3s6,* is located approximately 20 Mb from *Myc* on Chromosome 15, and four other genes, *Igk*, *Igl*, *Uros,* and *Actb,* are on Chromosomes 6, 16, 7, and 5, respectively. Since Chromosomes 12 and 15 are preferred neighbors in B cells [3], we considered the analysis of colocalization between *Eif3s6* and *Igh* to be of particular interest. If the high level of interchromosomal colocalization between *Myc* and *Igh* were simply due to the fact that the genes are on neighboring chromosomes, then we might expect *Eif3s6* and *Igh* to colocalize at similar frequencies when transcribed. We found that *Eif3s6* and *Igh* colocalize, but at significantly lower levels than *Myc*-*Igh*. Only 11% of *Eif3s6* signals colocalized with *Igh,* compared to approximately 25% for *Myc.* For the other genes we found that 9% of *Uros,* 8% of *Igk,* 6% of *Igl,* and 2% of *Actb* transcribing alleles colocalized with *Igh* (Figure 5). These considerably lower frequencies of co-association with *Igh* clearly demonstrate that *trans* co-association frequencies between different gene pairs can vary greatly. For example *Igh*-Myc trans colocalization is over 10-fold higher than *Igh*-*Actb,* indicating that the *Myc* and Igh trans colocalization frequency is statistically highly significant. However, the *Myc*-*Igh* co-association is also highly preferential, as indicated in the comparison between *Myc* and *Eif3s6*. *Myc* co-associates with *Igh* at a greater than 2-fold higher frequency than *Eif3s6*. This result demonstrates that not all genes on neighboring chromosomes co-associate at equal frequencies and shows that *Myc* and *Igh* preferentially co-associate in *trans*.

### *Myc* Alleles Move toward *Igh* upon Induction in B Cells

**Figure 6:**
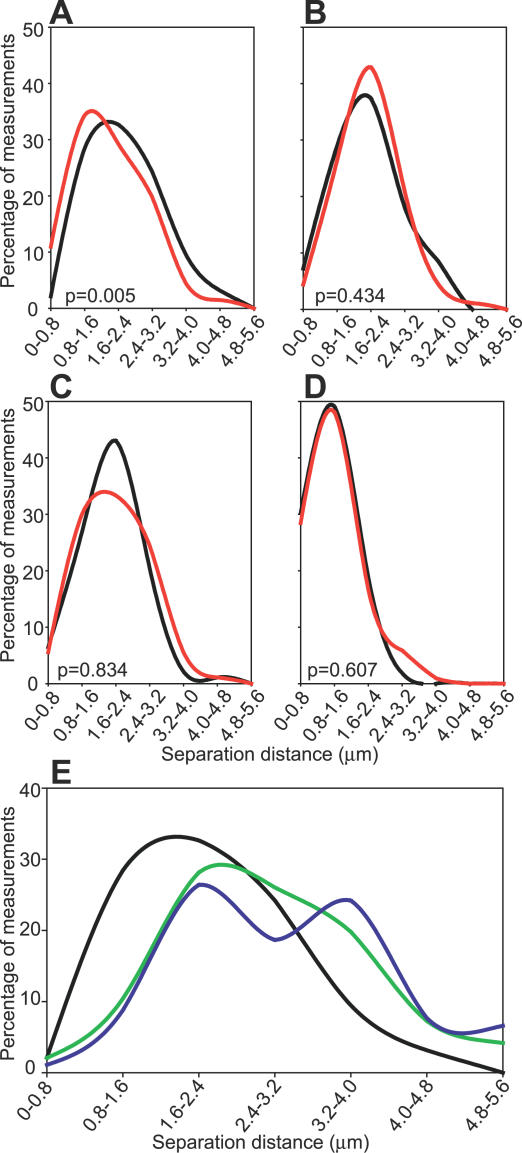
3D DNA FISH Measurements of Separation Distances (A–D) 3D DNA FISH separation distances between the *Igh* and the indicated alleles in unstimulated (black) and 5 min–stimulated (red) B cells. Values were grouped into 0.8 μm bins. Graphs show results for *Myc-Igh* (A), *Uros-Igh* (B), *Actb-Igh* (C), and *Fos-Igh* (D). At least 83 gene pairs were measured. *p*-Values show that *Myc* alleles are significantly shifted toward *Igh* upon stimulation. (E) Separation distance between *Igh* and *Myc* alleles in unstimulated B cells (black), kidney cells (green), and fetal liver erythroid cells (blue). *p*-Values for *Myc* and *Igh* separation distances for kidney and fetal liver versus unstimulated B cells are 3.3 × 10^−5^ and 6.2 × 10^−9^, respectively.

Our results suggest that 5 min after induction, many previously inactive *Myc* alleles are moving to preformed factories that contain transcriptionally active *Igh* alleles. If this interpretation is correct we would expect to see a net movement of *Myc* alleles toward *Igh* alleles upon stimulation of IE gene expression. To directly test this hypothesis we carried out 3D DNA FISH, measuring the separation distances between *Myc* and *Igh* alleles in unstimulated and stimulated cells. We found a statistically significant shift in the distribution of measurements upon B cell stimulation, changing from a mean separation distance of 2.16 μm to 1.83 μm (*p* = 0.005) (Figure 6A). This shows that across the population of cells *Myc* and *Igh* alleles are significantly closer together 5 min after induction. In contrast, we found no significant change in the distributions of measurements between *Igh* and two other genes in *trans, Actb* and *Uros* (Figure 6B and 6C). These results show there is no net movement between *Actb*-*Igh* and *Uros-Igh* upon B cell activation, indicating that these genes are not significantly changing their location relative to one another. However, there is net movement of *Myc* alleles toward *Igh* upon induction. We did not detect any net movement between *Igh* and *Fos* upon induction (Figure 6D), most likely because the range of separation between these physically linked genes is too small to detect subtle changes in relative positioning via light microscopy [9]. We conclude that increased *Myc* expression during IE gene induction involves the rapid relocation of *Myc* alleles to preassembled transcription factories, with many alleles migrating to a factory containing the *Igh* gene.

We also assessed separation distances between *Myc* and *Igh* alleles in two other tissue types, adult kidney and fetal liver erythroid cells. We detected much greater separation distances between *Myc* and *Igh* in these tissues compared to unstimulated B cells (Figure 6E). *Myc* and *Igh* were separated by an average of 2.78 and 3.20 μm in kidney and erythroid cells respectively, compared to an average of 2.16 μm in unstimulated B cells. These results are consistent with previously reported observations of tissue-specific positioning of genes [6,24] and chromosomes [3,25] and show that *Myc* and *Igh* are already in the same “nuclear neighborhood” in unstimulated B cells, which most likely facilitates their increased proximity and colocalization upon stimulation.

To corroborate the FISH results, we used the capturing chromosome conformation (3C) assay [9,26,27], which measures ligation frequency between in vivo formaldehyde cross-linked chromatin fragments. Ligation products were detected with four different primer pairs within the *Igh* and *Myc* loci in stimulated B cells (Figure S1). The primer pair that produced the most robust product (primer pair *d/g*) was used to detect ligation products in unstimulated and stimulated B cells. Over multiple experiments, ligation products were always detected in stimulated B cells, while in unstimulated cells the products were usually, but not always detected. In contrast, *Igh*/*Myc* ligation products were never detected in brain or kidney cells, indicating that juxtaposition of *Myc* and *Igh* is restricted to tissues in which both genes are expressed.

### Co-Association Frequencies of Translocation Partner Genes Are in Line with Their Relative Occurrences in Cancers

**Figure 7:**
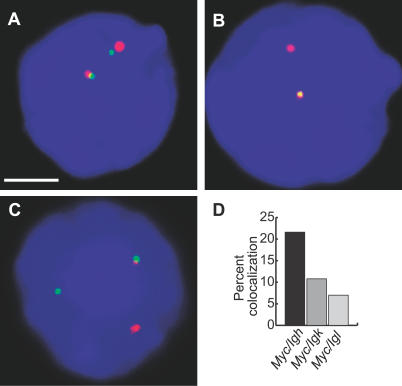
Co-Association Frequencies of Transcribing *Myc* Alleles with Immunoglobulin Genes (A–C) Double-label RNA FISH in stimulated B cells (10 min), showing *Myc* transcription signals in green, and in red, *Igh* (A), *Igk* (B), and *Igl* (C). DAPI staining is blue. Scale bar, 2 μm. (D) The percentage of *Myc* signals that overlap with *Igh, Igk,* or *Igl* signals. One hundred nuclei were examined in each case. *Myc*-*Igh* versus *Myc*-*Igk, p* = 0.02; versus *Myc*-*Igl, p* = 0.0003.

Importantly, *Myc* and *Igh* are the two most common translocation partners in Burkitt lymphoma and mouse plasmacytoma. Of these cancers 80% harbor *Myc*-*Igh* translocations, while the remaining cases contain *Myc*-*Igk* (15%) or *Myc*-*Igl* (5%) translocations [28,29]. To establish whether there is a relationship between the frequency of these translocations in plasmacytomas and the co-association frequencies of transcriptionally active alleles in normal B cells, we measured the extent to which the transcriptionally active *Myc* colocalized with *Igk* and *Igl* alleles in 10-min stimulated cells by RNA FISH. We found that 11% of transcribing *Myc* alleles colocalized with *Igk* and 7% colocalized with *Igl,* compared to 22% with *Igh* (Figure 7). Thus the frequencies of co-association between *Myc* and the immunoglobulin loci in transcription factories are in line with the appearance of their respective translocation frequencies in mouse plasmacytomas.

## Discussion

Our results show that IE gene induction involves the rapid nuclear relocation of previously inactive genes to preassembled transcription factories. This dynamic transcriptional organization is nonrandom and leads to the preferential juxtaposition of the *Myc* and *Igh* genes at transcription factories. Transcriptional colocalization may provide an opportunity and therefore an increased risk of illegitimate recombination resulting in a chromosomal translocation [30,31]. We cannot discount the possibility that differences in oncogenic potential result in selective outgrowth of one type of translocation versus another. However, it is striking that the co-association frequencies echo the appearance of specific translocations in plasmacytomas, suggesting that the juxtaposition frequency of specific genes in a transcription factory has a direct effect on their translocation frequency.

Upon B cell induction, signaling pathways converge upon the IE genes, a process that causes their relocation to transcription factories. Others have shown recently that upon activation, genes can undergo directed, actin and myosin-dependent relocalization, moving between 1 and 5 μm [32]. We cannot discount the possibility that similar forces may be involved in the relocation of genes to factories. However, we found that inactive *Myc* alleles are positioned on average 500 nm from the nearest factory, a distance that could conceivably be covered by random chromatin movements [33–35].

A key question concerns the basis of the preferred co-associations of specific genes in a common factory. The relative positions of genes in *cis* or on preferred neighbor chromosomes would be expected to affect the frequency of co-association in a factory [9] as it does recombination frequency [36]. On the other hand, it is possible that tissue-specific chromosomal positioning is driven by the net effect of thousands of preferential interchromosomal interactions between active (and inactive [37]) genes that serve as dynamic anchor points that facilitate chromosome positioning [38]. Preferential co-associations in factories may be the result of 3D spatial clustering of genes with related functions or genes coordinately regulated by common factors [9,37,39]. Genome-wide examination of the subsets of genes that preferentially co-associate may provide valuable information about these influences.

*Igh* translocations are presumed to occur through aberrant repair during programmed recombination, by recombinase activating gene protein (RAG) during V(D)J recombination, and activation-induced deaminase (AID) during somatic hypermutation and class switching [40]. Cleavage by RAG complexes at cryptic RAG recognition sites at other genes, and altered DNA structures have been implicated in the generation of some human *IGH* translocations [41,42]. However, the current consensus view is that cryptic RAG sites are not present in the major *Myc* breakpoint region. *Igh* translocations within the *Igh* diversity and joining regions occur in the bone marrow pre-B cells, which undergo V(D)J recombination [40]. However, most *Igh* translocations to *Myc* are found in the *Igh* class switch region and are believed to occur in germinal center B cells, the site of class switching [40]. There is strong evidence to suggest that genes may be susceptible to double-stranded breaks during transcription. The process of transcription creates considerable torsional stress [43], which can be relaxed by topoisomerases via introduction of transient double-stranded breaks. In fact, topoisomerase type IIβ-generated double-stranded breaks in the promoter regions of some genes have recently been shown to be required for regulated transcription [44]. Topoisomerase cleavage sites are common features of translocation hot spots [45]. Significantly, topoisomerase type IIβ binding sites have been mapped to the major breakpoint region of the human *MYC* gene, at the 5′ end of the first intron [46]. Further evidence of a link between transcriptional organization and recombination is suggested by two papers, one of which showed that actively transcribed yeast tRNA genes cluster in the nucleolus [47], and another which showed that recombination is higher between actively transcribed tRNA genes compared to inactive tRNA genes [48]. Interestingly, double-stranded break repair enzymes Ku70/80 are also associated with transcription factories [49]. In summary, the introduction and repair of double-stranded breaks may be commonplace in transcription factories. Therefore, interchromosomal co-associations between genes in factories may be expected to result in a heightened risk of aberrant repair of double-stranded breaks resulting in chromosomal translocations.

It is curious that evolution has allowed the interchromosomal juxtaposition of the *Myc* and *Igh* loci in transcription factories to persist, considering the potentially grave risks of such an organization. However, the apparent dangers of illegitimate recombinations may be outweighed by advantages of clustering transcribing genes, which may make efficient use of shared resources, or perhaps provide a degree of transcriptional coordination of subsets of genes.

## Materials and Methods

### B cell collection and stimulation.

CD43^−^ resting B cells were isolated from spleens of 6- to 8-wk-old BALB/c mice by magnetic cell sorting using CD43 microbeads (Miltenyi Biotec, http://www.miltenyibiotec.com) to deplete other cell types. Induction of B cells was done in PBS supplemented with 10 ng/ml recombinant mouse IL-4 (Stemcell Technologies, http://www.stemcell.com), 20 μg/ml purified rat anti-mouse monoclonal antibodies to CD40 (clone HM40–3, Serotec, http://www.serotec.com) and 10μg/ml goat anti-mouse IgM μ chain, F(ab′)2 fragment (Jackson Immunoresearch, http://www.jacksonimmuno.com) at room temperature for up to 15 min before fixation for FISH.

### RNA, DNA, and immuno-FISH.

RNA FISH was carried out as described previously [50,51]. We visualized *Igh* transcription with a dinitrophenol-labeled single-stranded DNA probe to the intronic enhancer region [19], followed by Texas Red detection. We prepared digoxygenin or biotin-labeled single-stranded DNA probes to detect *Fos, Myc, Eif3s6, Uros, Actb, Igk,* and *Igl,* intron sequences as described [19]. Primer sequences used to PCR-subclone the various probes are listed below. DNA FISH was carried out as previously described [52]. The following BAC clones (BACPAC Resources, http://bacpac.chori.org) were used: 234 kb RP23-98D8 for *Myc;* 178 kb RP24-233K8 for *Fos;* 166 kb RPCI24-258E20 for *Igh;* 151 kb RP24-132K17 for *Uros;* and 213 kb RP23-97O1 for *Actb*. For double-label experiments, we labeled one of the DNA FISH probes directly with AlexaFluor 594 and labeled the other probe with digoxigenin, detected with fluorescein-conjugated antibodies. Immunofluorescence and immuno-FISH was carried out as described [9,52], using a CTD4H8 antibody (Upstate Biotechnology, http://www.upstate.com) that was raised against a Ser5-phosphorylated CTD. This antibody is specific to phosphorylated forms of RNAP II [53].

Primers used to amplify RNA FISH probes were the following. *Fos* intron 1 sense, 5′-GCTTTGTGTAGCCGCCAGGT-3′; antisense 5′-AGAGGAAAGCGGAGGTGAGC-3′. *Fos* intron 2 sense, 5′-AAGTAGAGCTGGTGAGCAGCGATT-3′; antisense, 5′-AGAAAAGGACCAACATTCAGTTAAGG-3′. *Myc* intron 1 sense, 5′-AGCACAGATCTGGTGGTCTTTC-3′; antisense, 5′-CTCCTTCGAGCAGGGACTTAG-3′. *Myc* intron 2 sense, 5′-CTTCTCCACCACTCATTGGCATTA-3′; antisense, 5′-GGGAGGAAGTGGAAGATCACAGTT-3′. *Eif3s6* intron 1 sense, 5′-GTGAGGAAGCTTTGAGAAGGAGGA-3′; antisense, 5′-ATTAATTTTGCTGTTCCCTGCTGA-3′*. Uros* intron 6 sense, 5′-TCAGCGCCACAGCAAGGGTT-3′; antisense, 5′-GCCTTCCCTCCTTTGTTCCCAGT-3′*. Actb* intron 1 sense, 5′-TCGCTCTCTCGTGGCTAGTA-3′; antisense, 5′-TGGCGAACTATCAAGACACA-3′*. Igh* Iμ intron sense, 5′-AGCTGTGGCTGCTGCTCTTA-3′; antisense, 5′-AGCCTCGCTTACTAGGGCTCTC-3′*. Igl* J-C intron probe 1 sense, 5′-TGAGTGACTCCTTCCTCCTTTG-3′; antisense, 5′-TGGAGGCAGTGTGTAAAGTGTC-3′*. Igl* J-C intron probe 2 sense, 5′-GTTGTCTTGCAAGGGTCTTTTT-3′; antisense, 5′-GTGCGAATAAAAGAAGGGATTG-3′*. Igk* J-C intron sense, 5′-AAGACACAGGTTTTCATGTTAGGA-3′; antisense, 5′-AATAGAATTATGAGCAGCCTTTCC-3′.

### Microscopy and image analysis.

We examined RNA FISH signals on an Olympus BX41 epifluorescence microscope, and assessed 200 loci for each probe combination, except for *Uros*-*Igh,* for which 140 alleles were assessed. Transcription signals scored as colocalizing if the red and green signals overlapped to create a visible yellow signal. To assess the association of *Myc* DNA FISH signals and RNAP II foci, we captured image stacks of nuclei, using an Olympus BX41 epifluorescence microscope, equipped with a UPlanApo 100× oil objective to reduce chromatic aberration, and fitted with a motorized stage. Images were captured and analyzed using Analysis 3.2 image capture software, fitted with a RIDE module (SIS, http://www.sis.com). The stacks were deconvoluted using a nearest-neighbor algorithm with 85% haze removal, and analyzed. We analyzed 81 and 88 alleles in unstimulated and stimulated B cells, respectively. Statistical analysis was carried out using a two-sided Fisher exact test. For *Myc* alleles that were not co-associated with an RNAPII focus, we measured the separation distance from the edge of the gene signal to the edge of the nearest RNAP II immunofluorescence signal, and analyzed 27 alleles.

To measure the distances between *Igh* and genes in *trans* by DNA FISH, we collected image stacks using a Zeiss 510 Meta confocal microscope. Separation distances for each *Igh* allele and the nearest *Myc, Fos, Uros,* or *Actb* allele were measured on 3D-reconstructed image stacks using Volocity image analysis software (http://www.improvision.com/products/volocity). In all cases, we made measurements from center to center of the two gene signals. We analyzed at least 83 measurements for gene pairs in unstimulated and stimulated B cells, adult kidney cells, and E14.5 fetal liver cells. Changes in the distributions of measurements were assessed by two-sided Student *t*-test.

### Competitive RT-PCR.

The assay was carried out essentially as described [20]. RNA competitor fragments were generated by cloning 230 bp *Igh* and 236 bp *Fos* fragments that span 5′ exon-intron junctions. The plasmid containing the *Igh* fragment was digested with HindIII and AflII to release a 17 bp fragment, then religated. The *Fos* deletion was generated by BsmFI and NheI digestion to remove a 24 bp fragment. RNA was transcribed from linearized plasmids, then checked by gel electrophoresis and quantitated by UV spectrometry. Dilutions of controlled amounts of RNA was spiked into Trizol reagent that contained a known number of cells. RNA was extracted, reverse transcribed, and PCR amplified with nested primers.

To measure the numbers of primary transcripts over the length of the intron, the relative intensity of the endogenous RT-PCR product was compared to the spiked competitor RT-PCR product, measured using AIDA quantitation software. For *Igh,* the lanes with four copies of spiked competitor per cell was used for quantitation. For *Fos,* quantitation was obtained from the average from the lanes with 1 copy per cell and 0.5 copy per cell.

The primers used were as follows. *Igh*-f, 5′-CCTGGGAATGTATGGTTGTGGCTTC-3′; *Igh*-r, 5′-CCCCCTAAAGCAAT:GACTGAAGACTCA-3′; *Igh* nested-f, 5′-CCTCGGTGGCTTTGAAGGAACAAT-3′; *Igh* nested-r, 5′-CCCTAAAGCAATGACTGAAGACTCAGT-3′; *Fos*-f, 5′-AGCATCGGCAGAAGGGGCAAAGTA-3′; *Fos*-r, 5′-TGAAGTAGGAAGCTGTCAGGGAAACTG-3′; *Fos* nested-f, 5′-AGAAGGGGCAAAGTAGAGCAGGTGA-3′; *Fos* nested-r, 5′-TGTCAAAATCTGACAAGGGAGGGAAAG-3′.

### 3C assay.

We carried out the 3C assay as described previously [9]. We fixed B cells that had been stimulated for 5 min in 2% formaldehyde for 10 min at room temperature, and digested 1 × 10^6^ nuclei overnight with 600 units of BglII. We ligated digested chromatin (2 μg) with 2,000 units of T4 DNA ligase in a final volume of 800 μl. We cloned ligation product detected by primer pair *d*/*g* (see list below)and verified it by DNA sequencing. We tested the specificity of the 3C primers as previously described [9]. The primers used for 3C analysis were the following. *Myc a* forward, 5′-TCTACACCCCATACACCTCCA-3′; Myc a nested, 5′-CGAGAATATGCCATGAATTGG-3′. Myc b forward, 5′-GGGGAGGGAATTTTTGTCTATT-3′; Myc b nested, 5′-GGACAGTGTTCTCTGCCTCTG-3′. Myc c forward, 5′-TGCCCTCTCAGAGACTGGTAA-3′; Myc c nested, 5′-TTCCCCTTTCCTCTGTCATCT-3′. Myc d forward, 5′-ATTCTTCCAGGTGGTGATGTC-3′; Myc d nested, 5′-CTTCCCACAGCTCTCTTCCTT-3′. Igh e forward, 5′-AACCCATCTACCCATGTAGCC-3′; Igh e nested, 5′-CCTCTGACTGCCTCTTTTCCT-3′. Igh f forward, 5′-ACTGTGATCGGTTTTGGAGTG-3′; Igh f nested, 5′-CTGGGAGGGTTTGGTTCTTAC-3′. Igh g forward, 5′-CCCAGAACCTGAGAAGGAAGA-3′; Igh g nested, 5′-ACAGAACCGAACCATGACTTG-3′. Igh h forward, 5′-TTGGGCACTAAACACCACTTC-3′; Igh h nested, 5′-GGTGTGTGCAGGTTTTTGTCT-3′. *Hbb-b1* forward, 5′-CTCAGAGCAGTATCTTTTGTTTGC-3′; *Hbb-b1* nested, 5′-AGGATGAGCAATTCTTTTTGC-3′. *Calreticulin* Cal1, 5′-CTCCAGATAAACCAGTATGAT-3′; Cal2, 5′-AAACCAGATGAGGGCTGAAGG-3′. *Actb* Actb 1forward, 5′- CGGTGCTAAGAAGGCTGTTCC-3′; Actb 1nested, 5′- AGCAAGAGAGGTATCCTGACC-3′. *Actb* Actb 2forward, 5′- TGTGACAAAGCTAATGAGG-3′; Actb 2nested, 5′-TGAGTAGATGCACAGTAGG-3′.

## Supporting Information

Figure S1 3C Assay (807 KB PDF)

## Abbreviations

3C: capturing chromosome conformation
FISH: fluorescent in situ hybridization
IE: immediate early
RNAPII: RNA polymerase II
RT-PCR: reverse transcription PCR
