# Supplementary material for: Myc Dynamically and Preferentially Relocates to a Transcription Factory Occupied by Igh
Source: PLoS Biol. 2007 Jul 10;5(8):e192. doi: 10.1371/journal.pbio.0050192 (PMC1945077; doi:10.1371/journal.pbio.0050192)
Supplement: Figure S1 — (807 KB PDF) [file pbio.0050192.sg001.pdf]

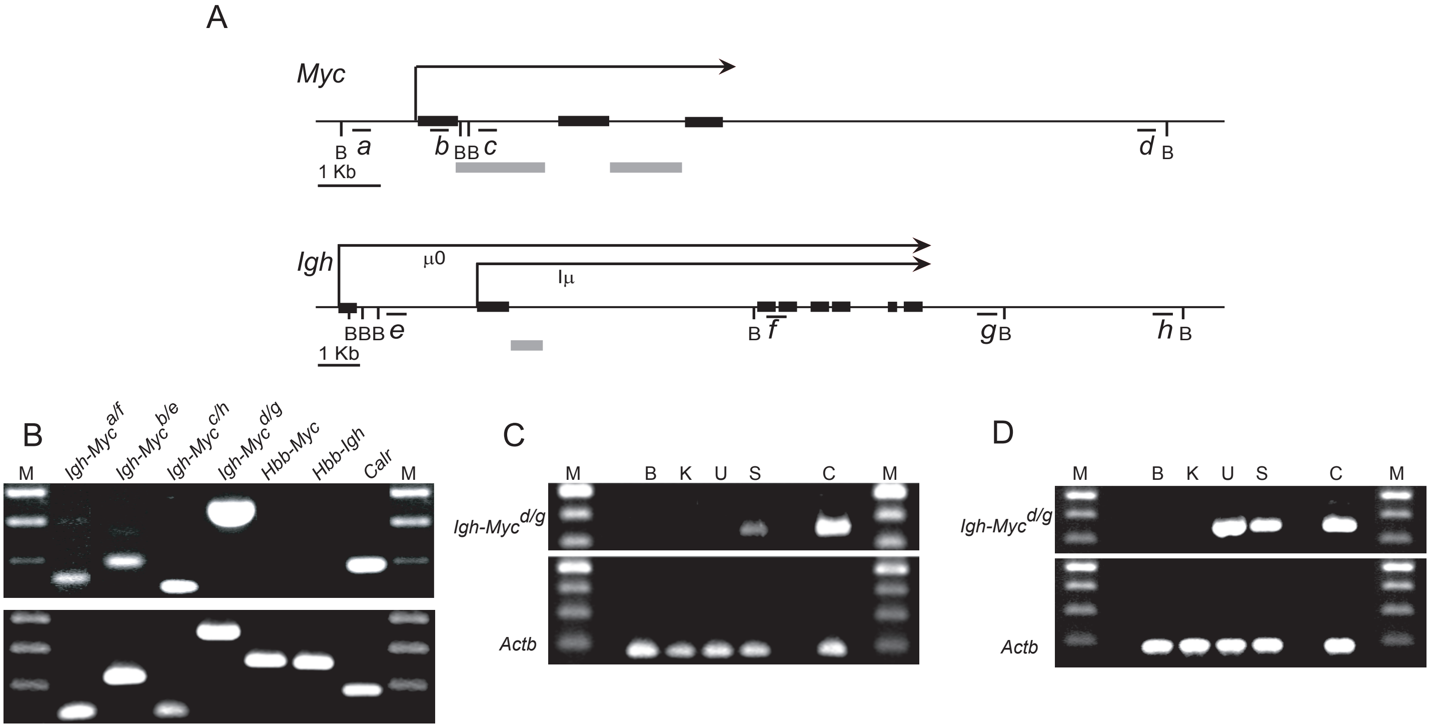

Supplementary Figure 1.

3C analysis. (A) Positions of primers used in 3C analysis and RNA FISH probes. Schematic map of the *Myc* gene and the constant region of the *Igh* gene. Positions of the *Myc*, μ0 and Iμ primary transcripts are shown as long arrows, exons are depicted as black bars. Locations of 3C primers (a-d in *Myc* and e-h in *Igh*) are shown. RNA FISH probes to *Myc* and *Igh* are shown as grey bars. B, BglII restriction sites. (B) Top panel, PCR detection of unique ligation products between *Igh* and *Myc* in stimulated B cells. Superscripts (a-h) denote the positions of primers within the *Igh* and *Myc* loci (panel a). All possible primer pair combinations were tested and only those that gave products of the correct size are shown. Note that different primer combinations gave products of varying intensities. No ligation was detected between *Hbb-b1* and *Myc* or *Igh*. Ligation between two adjacent restriction fragments of the ubiquitously expressed *Calr* or *Actb* genes were used as positive controls. PCR control products were detected in equi-molar mixtures of all fragments ligated at random (bottom panel). The primer pair that yielded the most robust product (*d/g*) was used for further analysis (panels C and D). Over several experiments, ligation products were always detected between *Igh* and *Myc* in stimulated B cells (S). Ligation products between *Igh* and *Myc* in unstimulated B cells (U) were detected in some but not all experiments. Ligation products between *Igh* and *Myc* were never detected in brain (B) or kidney (K) cells. PCR control products were detected in equi-molar mixtures of all fragments ligated at random (C). M, DNA size marker. PCR products were verified by DNA sequencing.
